# Serum levels of anti-PF4 IgG after AZD1222 (ChAdOx1 nCoV-19) vaccination

**DOI:** 10.1038/s41598-022-11623-9

**Published:** 2022-05-13

**Authors:** Taylor S. Cohen, Elizabeth J. Kelly, Sven Nylander, Himanshu Bansal, Brett M. Jepson, Prakash Bhuyan, Magdalena E. Sobieszczyk, Ann R. Falsey

**Affiliations:** 1 Microbiome Discovery, Vaccines and Immune Therapies, BioPharmaceuticals R&D, AstraZeneca, Gaithersburg, MD 20878 USA; 2 Translational Medicine, Vaccines and Immune Therapies, BioPharmaceuticals R&D, AstraZeneca, Gaithersburg, MD USA; 3 Clinical Development, Vaccines and Immune Therapies, Biopharmaceuticals R&D, AstraZeneca, Gothenburg, Sweden; 4 Biometrics, Vaccines and Immune Therapies, BioPharmaceuticals R&D, AstraZeneca, Gaithersburg, MD USA; 5 Vaccines and Immune Therapies, BioPharmaceuticals R&D, AstraZeneca, Gaithersburg, MD USA; 6 Division of Infectious Diseases, Department of Medicine, Columbia University Irving Medical Center and New York-Presbyterian Hospital, New York, NY USA; 7 University of Rochester Medical Center, Rochester, NY USA

**Keywords:** Immunology, Vaccines, DNA vaccines

## Abstract

Rare cases of thrombosis with thrombocytopenia syndrome (TTS) have been reported after AZD1222. Anti-platelet factor-4 (PF4) antibodies were observed in patients following presentation of TTS, however it is unclear if AZD1222 was responsible for inducing production of anti-PF4. Paired samples (baseline and day-15) from a phase 3 trial of AZD1222 vs placebo were analyzed for anti-PF4 levels; 19/1727 (1.1%, AZD1222) vs 7/857 (0.8%, placebo) participants were anti-PF4-IgG-negative at baseline but had moderate Day-15 levels (*P* = 0.676) and 0/35 and 1/20 (5.0%) had moderate levels at baseline but high Day-15 levels. These data indicate that AZD1222 does not induce a clinically relevant general increase in anti-PF4 IgG.

## Introduction

Following the utilization of COVID-19 vaccines, rare cases of thrombosis with thrombocytopenia syndrome (TTS) have been reported^1–4^. Publications have noted that individuals experiencing TTS after receiving AZD1222 (ChAdOx1 nCoV-19), which has been reported primarily following the first dose and as generally occurring within 2 weeks post vaccination^2,5^, had elevated levels of antibodies targeting platelet factor 4 (PF4)^1–3^. While these studies point to a strong association between anti-PF4 IgG and TTS, they do not address whether AZD1222 causes a generalized increase in antibody levels post administration. To determine if vaccination with AZD1222 induces an increase in levels of anti-PF4 IgG, we analyzed paired serum samples collected prior to and 15 days after vaccination with AZD1222 or placebo from participants in a multicenter, randomized Phase 3 study^6^.

## Results

Serum samples were analyzed from 1777 participants who received AZD1222 and 888 who received placebo. Key demographics and clinical characteristics of these participants are summarized in Supplementary Table S1. None of the 2665 participants experienced TTS following administration of vaccine or placebo. Paired serum samples were available from 1762 participants who received AZD1222 and 877 who received placebo.

**Figure 1:**
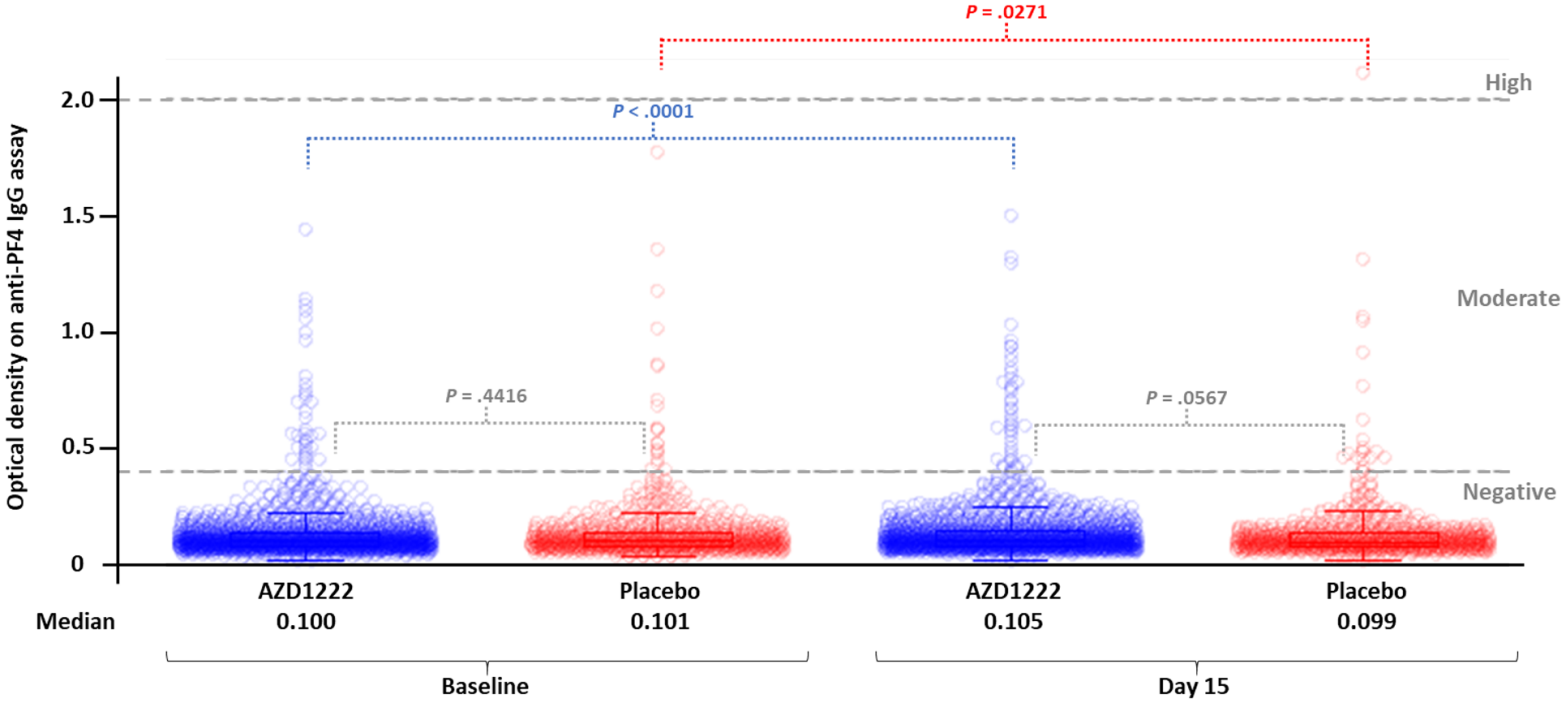
Individual values and summary statistics for optical density from the anti-PF4 IgG assay prior to administration of AZD1222 or placebo and at Day 15. Baseline samples were collected on or before the first dose of AZD1222 or placebo, and Day 15 samples were collected between Day 8 and Day 22 (± 7 day window). The bottom and top edges of the boxes within each collection of optical density data points indicate the first and third quartiles, the lines inside the boxes are the medians, and the markers inside the boxes are the means. Any points > 1.5 × IQR from the box are considered outliers. Whiskers indicate the minimum and maximum optical density values after removing outliers. P-values comparing treatment arms at Baseline and at Day 15 are based on a 2-sample t-test. P-values comparing Baseline and Day 15 within a treatment arm are based on a paired t-test.

In the AZD1222 and placebo groups, respectively, 98.0% and 97.7% of baseline serum samples were classified as being negative for anti-PF4 IgG, as were 97.5% and 97.6% of Day 15 serum samples (Table 1). Optical density (OD) assay values were similar between the AZD1222 and placebo group at both baseline (median OD: 0.100 and 0.101, respectively; *P* = 0.4416) and Day 15 (median OD: 0.105 and 0.099, respectively; *P* = 0.0567) (Fig. 1). Overall, 96.9% of paired samples in both groups (AZD1222: 1708/1762; placebo: 850/877) were classified as negative at baseline and also negative at Day 15. There was a minimal increase in OD values in the AZD1222 arm from baseline to Day 15 (median OD: 0.100–0.105; *P* < 0.0001); however, these median values fall below the threshold for positivity, and there was no difference between the AZD1222 and placebo groups in the proportion of individuals shifting from negative at baseline to moderate or high at Day 15 (1.1% vs 0.8%; *P* = 0.676). At baseline, 2.0% and 2.3% of individuals in the AZD1222 and placebo groups, respectively, had samples positive for anti-PF4 IgG (OD > 0.4); all were classified as moderate. None of these individuals in the AZD1222 group increased to a high level of anti-PF4 IgG at Day 15 compared to 1 in the placebo group, while 10/35 (28.6%) and 6/20 (30.0%), respectively, decreased below the threshold for positivity.

**Table 1:** Anti-PF4 antibody levels at baseline, prior to administration of AZD1222 or placebo, and at day 15.

Baseline		Day 15 level^a^, no. (%) of participants		
Level^a^	No. (%) of participants	Negative	Moderate	High
**AZD1222 group**				
Negative	1727 (98.0)	1708 (98.9)	19 (1.1)^b^	0
Moderate	35 (2.0)	10 (28.6)	25 (71.4)	0
High	0	0	0	0
Total	1762 (100)	1718 (97.5)	44 (2.5)	0
**Placebo group**				
Negative	857 (97.7)	850 (99.2)	7 (0.8)^b^	0
Moderate	20 (2.3)	6 (30.0)	13 (65.0)	1 (5.0)
High	0	0	0	0
Total	877 (100)	856 (97.6)	20 (2.3)	1 (0.1)

The denominators for percentages in the Baseline column are the total numbers of participants within each treatment group with data at Baseline and Day 15. The denominators for percentages in the Negative, Moderate, and High columns at Day 15 are the numbers in the Baseline column for the respective rows.

^a^Anti-PF4 IgG antibody level was classified according to optical density (OD) value on Immucor assay as negative (OD ≤ 0.4), moderate (OD > 0.4 to ≤ 2.0), or high (OD > 2.0). Baseline samples were collected on or before the first dose of AZD1222 or placebo, and Day 15 samples were collected between Day 8 and Day 22 (± 7 day window).

^b^*P* = 0.676. P-value is based on a Fisher’s Exact test, comparing treatment arms in terms of the total proportion of participants increasing from negative at baseline to moderate or high at Day 15. Participants who had moderate or high values at baseline are excluded from the statistical comparison.

## Discussion

These data fill a critical gap in our understanding of anti-PF4 IgG in individuals vaccinated with AZD1222. Our analysis demonstrates that AZD1222 did not result in an increased rate of detection of anti-PF4 IgG post-vaccination compared to placebo during the period of highest TTS risk. The majority of Day 15 samples that were classified as moderate were from participants with a moderate level of anti-PF4 IgG at baseline, and the only Day 15 sample classified as high was from a participant in the placebo group with a moderate level at baseline. The increase in median OD in the AZD1222 group from 0.100 at baseline to 0.105 at Day 15, although statistically significant, did not represent a clinically meaningful increase, with the median remaining well below the positive threshold. Our data are supported by findings from a previous analysis of a smaller cohort of patients who received AZD1222 or BNT162b2, which also reported low-titer anti-PF4/polyanion IgG post-vaccination primarily in individuals who were seropositive pre-vaccination^10^. A similarly low rate of anti-PF4/polyanion IgG post-vaccination was seen in a cross-sectional study of Thai healthcare workers who received AZD1222 or CoronaVac^11^; however, pre-vaccination data were not available. All positive sera had low titers, and none induced platelet aggregation^11^.

Additionally, our data indicate a ‘background’ prevalence rate of anti-PF4 IgG of 2.0–2.3%. This reflects the detection rates seen with other commercial immunoassays detecting IgG, IgA, and IgM in a healthy population^12^. The rate also reflects the low prevalence reported in the general population, which is estimated to be 3–8%^13,14^ but is dependent on population characteristics, as well as the very low prevalence of anti-PF4 IgG associated with heparin-induced thrombocytopenia in healthy individuals^15^. Our current understanding of anti-PF4 IgG levels in TTS is based on retrospective analyses following clinical presentation^1–4^, which have therefore been unable to assess antibody levels prior to syndrome manifestation. A limitation of our findings is that no TTS events were observed in this clinical trial of AZD1222^6^, and therefore no TTS events are captured by this analysis. The identification of specific markers associated with the development of TTS is challenging due to the extremely rare frequency of TTS^5^.

In conclusion, these data support that vaccination with AZD1222 does not result in a generalized increase in anti-PF4 IgG levels in vaccine recipients.

## Methods

Full study methodology has been reported previously^6^. Briefly, this phase 3, double-blind, placebo-controlled trial conducted in the United States, Chile, and Peru (ClinicalTrials.gov number NCT04516746; First posted 18/08/2020) assessed the efficacy, immunogenicity, and safety of two intramuscular doses of AZD1222 (N = 21,635) or saline placebo (N = 10,816; 2:1 randomization) given 4 weeks apart in participants aged ≥ 18 years who were at increased risk of SARS-CoV-2 infection. As part of an immunogenicity sub-study, serum samples were obtained from participants on days 1 (prior to first dose) and 15 (post first dose). The trial was conducted in accordance with the principles of the Declaration of Helsinki and the International Council for Harmonisation Good Clinical Practice guidelines. The trial protocol (available with the full text of the primary report^6^) and amendments were approved by the following independent ethics committees (IECs)/institutional review boards (IRBs): Universidad de Chile – Facultad de Medicina (covering 3 centers in Chile), El Comite Nacional Transitoria de Etica en Investigacion Para la Evaluacion y Supervision Etica de los Ensayos Clinicos de la Enfermedad (covering 3 centers in Peru), WCG IRB (covering 77 centers in the United States), Oregon Health & Science University, Sutter Health Institutional Review Board, The University of Vermont Committees on Human Subjects, The Ohio State Biomedical Sciences Institutional Review Board, and Columbia University. Prior to enrolment, all participants provided either written or secure electronic informed consent. The datasets generated and/or analyzed during the current study are not publicly available due to the study being ongoing at the time of analysis, but are available from the corresponding author on reasonable request.

For the purposes of this exploratory, post-hoc analysis, all participants in the immunogenicity sub-study with available serum samples obtained on both day 1 and day 15 were included, and their paired serum samples were assessed for anti-PF4 IgG using a validated IgG-specific PF4-polyvinylsulfate enzyme-linked immunosorbent assay (Immucor) similar to that previously used^1^. The assay output is an OD value that correlates with the level of anti-PF4 IgG. Serum samples were classified as being negative (OD ≤ 0.4, the threshold for a positive anti-PF4 IgG reading as defined by the manufacturer) for anti-PF4 IgG, or as having moderate (OD > 0.4 to ≤ 2.0) or high (OD > 2.0) levels of anti-PF4 IgG. This threshold for classification of high anti-PF4 IgG is based on the majority of TTS case reports having an OD of greater than 2.0 for anti-PF4 IgG, as highlighted in the American Society of Hematology TTS guidelines^7^, and the strong correlation between an OD of > 2.0 and heparin-induced thrombocytopenia^8,9^. The proportion of participants in whom the baseline (Day 1, pre-vaccination) serum sample was classified as negative and who had a Day 15 sample classified as moderate or high was compared between the AZD1222 and placebo groups using a Fisher’s Exact test; overall differences in median OD values were compared between baseline and Day 15 within each group and between the AZD1222 and placebo groups at baseline and Day 15 using t-tests.

## Supplementary Information

Supplementary Table 1.

## Data Availability

Data underlying the findings described in this manuscript may be obtained in accordance with AstraZeneca’s data sharing policy described at https://astrazenecagrouptrials.pharmacm.com/ST/Submission/Disclosure.
