# Supplementary material for: Serum levels of anti-PF4 IgG after AZD1222 (ChAdOx1 nCoV-19) vaccination
Source: Sci Rep. 2022 May 13;12:7961. doi: 10.1038/s41598-022-11623-9 (PMC9103599; doi:10.1038/s41598-022-11623-9)
Supplement: Supplementary file 1 — Supplementary Table 1. [file 41598_2022_11623_MOESM1_ESM.docx]

**Supplementary Information**

**Serum levels of anti-PF4 IgG after AZD1222 (ChAdOx1 nCoV-19) vaccination**

**Authors:**

Taylor S. Cohen*^1^, Elizabeth J. Kelly^2^, Sven Nylander^3^, Himanshu Bansal^4^, Brett M. Jepson^4^, Prakash Bhuyan^5^, Magdalena E. Sobieszczyk^6^, and Ann R. Falsey^7^

**Author affiliations:**

^1^Microbiome Discovery, Vaccines and Immune Therapies, BioPharmaceuticals R&D, AstraZeneca, Gaithersburg, MD, USA; ^2^Translational Medicine, Vaccines and Immune Therapies, BioPharmaceuticals R&D, AstraZeneca, Gaithersburg, MD, USA; ^3^Clinical Development, Vaccines and Immune Therapies, Biopharmaceuticals R&D, AstraZeneca, Gothenburg, Sweden; ^4^Biometrics, Vaccines and Immune Therapies, BioPharmaceuticals R&D, AstraZeneca, Gaithersburg, MD, USA; ^5^Vaccines and Immune Therapies, BioPharmaceuticals R&D, AstraZeneca, Gaithersburg, MD, USA; ^6^Division of Infectious Diseases, Department of Medicine, Columbia University Irving Medical Center and New York-Presbyterian Hospital, New York, NY, USA; and ^7^University of Rochester Medical Center, Rochester, NY, USA

***Corresponding author:**

Taylor S. Cohen, Microbiome Discovery, Vaccines and Immune Therapies, BioPharmaceuticals R&D, AstraZeneca, Gaithersburg, MD, 20878 USA
email: [taylor.cohen@astrazeneca.com](mailto:taylor.cohen@astrazeneca.com); phone: 301-398-2405.

**Supplementary Table 1. Baseline demographics and clinical characteristics of participants in the phase 3 trial From whom serum samples were analyzed**

|  | **AZD1222 (n = 1777)** | **Placebo (n = 888)** |
| --- | --- | --- |
| Demographics |  |  |
| Age, median (range), y | 55.0 (18.0–94.0) | 55.0 (18.0–90.0) |
| Age group, no. (%) |  |  |
| ≥18 – <56 y | 906 (51.0) | 450 (50.7) |
| ≥56 – <70 y | 461 (25.9) | 225 (25.3) |
| ≥70 y | 410 (23.1) | 213 (24.0) |
| Sex, no. (%) |  |  |
| Male | 1072 (60.3) | 508 (57.2) |
| Female | 705 (39.7) | 380 (42.8) |
| Race |  |  |
| White | 1580 (88.9) | 789 (88.9) |
| Black or African American | 94 (5.3) | 55 (6.2) |
| Asian | 44 (2.5) | 16 (1.8) |
| Other/multiple | 38 (2.1) | 19 (2.1) |
| Not reported/unknown | 21 (1.2) | 9 (1.0) |
| Clinical characteristics |  |  |
| Comorbidities at baseline, no. (%)^a^ | 1109 (62.4) | 566 (63.7) |
| Serum samples for analysis, no. (%) |  |  |
| Paired baseline and Day 15 | 1762 (99.2) | 877 (98.8) |
| Baseline only | 15 (0.8) | 10 (1.1) |
| Day 15 only | 0 | 1 (0.1) |

^a^Comorbidities are per the list of coexisting conditions in the primary publication.^6^
